# Urolithin A alleviates cell senescence by inhibiting ferroptosis and enhances corneal epithelial wound healing

**DOI:** 10.3389/fmed.2024.1441196

**Published:** 2024-09-16

**Authors:** Xiao-Xiao Guo, Xue-Jiao Chang, Qi Pu, Ao-Ling Li, Jing Li, Xin-Yu Li

**Affiliations:** Department of Ophthalmology, Tongji Hospital, Tongji Medical College, Huazhong University of Science and Technology, Wuhan, China

**Keywords:** Urolithin A, wound healing, corneal epithelial cell, cell senescence, ferroptosis

## Abstract

**Purpose:**

To analyze the therapeutic effect and mechanism of Urolithin A (UA) on delayed corneal epithelial wound healing.

**Methods:**

The C57BL/6 mice were continuously exposed to hyperosmotic stress (HS) for 7 days followed by the removal of central corneal epithelium to establish a delayed corneal epithelial wound healing model *in vivo*. *In vitro*, the human corneal epithelial cell line (HCE-T) was also incubated under HS. UA was administered *in vivo* and *in vitro* to study its effects on corneal epithelial cells. Senescence-associated β-galactosidase (SA-β-gal) staining was performed to detect the level of cell senescence. Transcriptome sequencing (RNA-seq) was conducted to elucidate the molecular mechanism underlying the effect of UA on corneal epithelial repair. Additionally, the expression of senescence-related and ferroptosis-related genes and the levels of lipid peroxides (LPO) and malondialdehyde (MDA) were measured.

**Results:**

Hyperosmotic stress (HS) significantly increased the proportion of SA-β-gal staining positive cells in corneal epithelial cells and upregulated the expression of p16 and p21 (*p* < 0.0001). Topical application of UA decreased the accumulation of senescent cells in corneal epithelial wounds and promoted epithelial wound healing. The results of RNA-seq of HS-induced corneal epithelial cells showed that the ferroptosis pathway was significantly dysregulated. Further investigation revealed that UA decreased the level of oxidative stress in HCE-T cells, including the levels of LPO and MDA (*p* < 0.05). Inhibition of ferroptosis significantly prevented cellular senescence in HS-induced HCE-T cells.

**Conclusion:**

In this study, UA promoted HS-induced delayed epithelial wound healing by reducing the senescence of corneal epithelial cells through the inhibition of ferroptosis.

## 1 Introduction

The corneal epithelium acts as a physical barrier and prevents the entry of external substances into the eye. The integrity and health of the corneal epithelium largely depend on the normal healing of the corneal epithelium after injury (1, 2). Lesions can damage the corneal epithelium and disrupt its barrier function. Although the corneal epithelium usually heals quickly, some pathological conditions impair or delay the healing of the corneal epithelium. Studies have shown that sustained hyperosmotic stress (HS) can induce cell cycle arrest, apoptosis, and DNA damage (3, 4). For example, corneal epithelial cells in patients with severe dry eye and diabetes remain in the HS state for a long time, resulting in corneal epithelial cell damage and a decrease in its healing ability; moreover, complications such as recurrent epithelial defects or persistent corneal erosion may occur (5, 6).

Cell senescence is induced by various stimuli and stress, including oxidative stress, DNA damage, metabolic damage, and activation of oncogenes (7–9). Studies have found that high accumulation of senescent cells may drive delayed wound healing (10, 11). Alleviating hyperglycemia-induced cell senescence can accelerate diabetic wound healing (12), and the removal of senescent cells can promote wound healing and delay age-related diseases (13–15). Therefore, targeting cellular senescence is a novel approach to treating chronic poor wound healing.

Urolithin A (UA) is a metabolite produced by gut microbes acting on ellagic acid and ellagitannin, which are found in pomegranates, berries, and nuts (16). UA has anti-inflammatory, antioxidant, anti-tumor, and anti-aging effects (17–19). Some studies have shown that UA can alleviate oxidative stress-induced senescence of nucleus pulposus cells and ultraviolet radiation A-induced senescence of human dermal fibroblasts (20–22). As UA also prevents auditory cell senescence, it is a promising agent for treating age-related hearing loss (23). However, the effect of UA on corneal epithelial cell senescence is unclear. Therefore, we investigated whether UA can alleviate corneal epithelial cell senescence in chronic wound healing and elucidated its mechanism of action.

## 2 Materials and methods

### 2.1 Animal model

In total, male C57BL/6 mice (*n* = 83; 6–8 weeks old) were purchased from Bernd Laboratory Animal Technology Co., Ltd. (Hubei, China). The mice were divided into the control, HS, and HS + UA groups. Corneas of HS group and HS + UA group were treated with 500 mOsm/L NaCl solution by eye drops 10 times a day for 7 days. After the last intervention, the corneal epithelium was scraped. Mice in the control group were administered physiological saline. Mice in the HS + UA group were administered subconjunctival injection of UA (5 μL, 10 mmol/L) every other day for five consecutive times since the beginning of the experiment. The mice in the HS group and the control group were injected with an equal volume of physiological saline. AlgerBrush II rust remover (Alger, Lago Vista, TX, United States) was used to remove the epithelium within 2.5 mm of the central cornea under anesthesia. The mouse cornea was harvested for subsequent experimental analysis at predetermined time points.

### 2.2 Evaluating the damage to the ocular surface

The healing time of the corneal epithelium in the C57BL/6 mice was recorded after de-epithelialization, and the corneal epithelial defect area was compared 0, 18, 24, 40, 48, and 64 h after surgery. The cornea was stained with 0.1% sodium fluorescein, and the degree of healing of the corneal epithelium was observed and photographed using a hand-held slit lamp. The area of corneal epithelial defect was quantified by the ImageJ software (National Institutes of Health, United States).

### 2.3 Hematoxylin and eosin staining

At the 18th hour after the epithelium was removed, eyeballs of mice from different groups were harvested, fixed, dehydrated with 70–99% ethanol, and then, embedded in paraffin. The sections (5 μm thick) were stained with hematoxylin and eosin (H&E) to assess the degree of pathological alteration.

### 2.4 Immunofluorescence staining

For immunofluorescence staining, corneal tissue sections or cells were blocked with a 10% donkey serum solution (Boster Biological Technology, United States) for 30 min. Then, the samples were incubated with primary antibodies overnight at 4°C. After incubation, the samples were washed twice with PBS, incubated with the corresponding source of secondary antibodies for 45 min, and washed twice with PBS again. The nuclei were stained with 10 μg/mL DAPI and analyzed. Images were captured under a fluorescence microscopy (Olympus, Japan). The quantitation was performed using the ImageJ software.

### 2.5 Immunohistochemical staining

Paraffin sections underwent immunohistochemical (IHC) staining following standard protocols. Antigen unmasking was required for paraffin sections and was performed using Tris-EDTA buffer (pH 9.0). The tissue endogenous peroxide activity was quenched using a blocking solution (3% hydrogen peroxide). The slides were incubated with 10% goat serum for 45 min. After incubation, excess serum was removed from the slides, and the primary antibodies against p16 (#bs-0740R, Bioss, China, 1:300), p21 (#28248-1-AP, Proteintech, China, 1:200), and p53 (#10442-1-AP, Proteintech, China, 1:300) were added and incubated at 4°C overnight. After washing with 1 × PBS buffer, the secondary antibodies were added and incubated with DAB and a substrate color development system. Then, the sections were observed and examined under an optical microscope.

### 2.6 Cell culture and treatments

The human corneal epithelial (HCE-T) cells were purchased from MeisenCTCC (Zhejiang, China). The HCE-T cells were cultured in plates in a humidified atmosphere containing 5% carbon dioxide at 37°C. Dulbecco’s modified Eagle’s medium/F12 containing 5 μg/mL insulin, 10 ng/mL human epidermal growth factor, 10% fetal bovine serum, and 1% penicillin/streptomycin were used as the culture medium. The hypertonic state was achieved by adding NaCl to the medium. The HCE-T cells were cultured under different hypertonic conditions (400 mOsm/L, 450 mOsm/L, and 500 mOsm/L) for 120 h.

### 2.7 Cell viability and LDH assay

Cell viability was determined by the Cell Counting Kit 8 assay (CCK-8, #C0037, Beyotime, China), following the manufacturer’s guidelines. The HCE-T cells were inoculated in 96-well plates at a density of 5,000 cells/well and cultured in the cell incubator at 37°C with 5% CO_2_. After the cells were attached to the wall, different concentrations of UA were added, followed by treatment for 24 h and 48 h. The cells were further treated with 10 μL of CCK-8 solution for 1–4 h, and the optical density was measured at 450 nm by enzyme-labeler to obtain the OD values.

Cytotoxicity was measured by the lactate dehydrogenase (LDH) release assay. Extracellular LDH release was determined by the LDH cytotoxicity detection kit (#C0016, Beyotime, China), following the manufacturer’s instructions.

### 2.8 Cell scratch test

The HCE-T cells were inoculated in six-well plates and incubated overnight at 37°C. When the cell fusion reached about 90%, the medium was discarded and a 200 μL pipette tip was used to scratch the bottom of the plate. The scattered cells were washed thrice with PBS. According to the group, the corresponding medium and drugs were added for intervention, and incubation was continued at 37°C for 24 h. The cells that migrated to the labeled reference area to repair the wound were photographed, and the images were quantified using the ImageJ software. Wound healing was assessed by calculating the ratio of the difference between the original and remaining wound areas at 24 h.

### 2.9 Senescence-associated β-galactosidase activity assay

The senescence-associated β-galactosidase (SA-β-gal) staining kit (Beyotime, China) was used to stain the corneal patch or HCE-T cells *in situ*, following the manufacturer’s instructions, to detect the senescence status of the cells. Briefly, mouse corneas or cells were washed thrice with PBS. Then, β-galactosidase fixing solution was added to fix the cells at room temperature for 15 min, followed by washing with PBS three times. Subsequently, 1 mL of β-galactosidase staining solution was added and incubated overnight in a biochemical incubator at 37°C. Next, the dye working solution was discarded. SA-β-gal-positive cells appeared blue, and the number of positive-stained cells per 200 cells in a randomly selected field of view was calculated under an optical microscope.

### 2.10 RNA sequencing

To sequence cellular RNA, a high-throughput sequencing service (Dancheng Biotechnology, Shanghai, China) was used. The total RNA of HCE-T cells was extracted using Trizol, according to the instructions, and the mRNA was enriched by magnetic beads and Oligo (dT). The RNA concentration was determined using the Nanodrop 2000 spectrophotometer (Thermo Fisher Scientific). RNA was sequenced using the HiSeq 2000 system (Illumina, San Diego, CA, United States). Subsequently, *p* < 0.05 and fold change >2 generated by DESeq2 were analyzed for the enrichment of biological terms with the Database for Annotation, Visualization, and Integrated Discovery (DAVID) bioinformatics platform. The gene expression patterns were analyzed using volcano plots. The Kyoto Encyclopedia of Genes and Genomes (KEGG) pathway enrichment analysis of the differentially expressed transcripts was performed using R based on the hypergeometric distribution.

### 2.11 Measurement of intracellular iron and lipid peroxidation

Labile intracellular iron was measured using the calcein acetoxymethyl (AM) ester quenching method (#C2012-0.1 mL, Beyotime). The HCE-T cells were washed with 1× PBS, treated with calcein AM (1 μM), and incubated at 37°C for 30 min. The culture medium was replaced with a fresh medium and incubated for 30 min to ensure that calcein AM was fully hydrolyzed by lactase to generate calcein with green fluorescence. Finally, the cells were washed thrice with PBS and observed under a fluorescence microscope (Olympus, Japan).

Lipid peroxidation (LPO) in HCE-T cells was detected by the BODIPY 581/591 C11 (BODIPY C11) probe (#D3861, Thermo Fisher Scientific, United States). The HCE-T cells on the slide were incubated with BODIPY-C11 (2 μM) in the dark for 30 min. After incubation, the working solution was discarded, and the cells were washed with 1 x PBS buffer twice for 5 min each time. Imaging was performed using a fluorescence microscope (Olympus, Japan).

### 2.12 Measurement of malondialdehyde and glutathione

The malondialdehyde (MDA) content in HCE-T cells was determined using the MDA assay kit (#E-BC-K028-M, Elabscience, China), following the manufacturer’s instructions. The oxidized glutathione (GSSG) and reduced glutathione (GSH) levels were estimated using a Total GSH/GSSG Colorimetric Assay Kit (#E-BC-K097-M, Elabscience, China).

### 2.13 Reverse transcription and quantitative real-time polymerase chain reaction

Total RNA was extracted from HCE-T cells with the RNeasy Mini Kit (Qiagen, Valencia, CA, United States), following the manufacturer’s instructions. In total, 1–2 μg of total RNA was reverse-transcribed to cDNA using the cDNA Transcription Kit (Vazyme, United States). Reverse transcription and quantitative real-time polymerase chain reaction (RT-qPCR) were performed in a 20 μL solution containing cDNA, TaqMan Gene Expression Assay Mix, and a universal PCR Master Mix (Vazyme, United States). The expression of the target gene was normalized to that of Gapdh. The cDNA was amplified using the following primers: p16, forward, 5′-GGGTTTTCGTGGTTCACATC-3′, reverse, 5′-CTAGACGCTGGCTCCTCAGTA-3′; p21, forward, 5′-CGATGGAACTTCGACTTTGTCA-3′, reverse, 5′-GCACAAGGGTACAAGACAGTG-3′; p53, forward, 5′-TGAAGCTCCCAGAATGCCAG-3′, reverse, 5′-CAGTCAGAGCCAACCTCAGG-3′; Acyl-CoA synthetase long chain family member 4 (ACSL4), forward, 5′-CATCCCTGGAGCAGATACTCT-3′, reverse, 5′-TCACTTAGGATTTCCCTGGTCC-3′; ferritin heavy chain 1 (FTH1), forward, 5′-CCCCCATTTGTGTGACTTCAT-3′, reverse, 5′-GCCCGAGGCTTAGCTTTCATT-3′; glutathione peroxidase 4 (GPX4), forward, 5′-GAGGCAAGACCGAAGTAAACTAC-3′, reverse, 5′-CCGAACTGGTTACACGGGAA-3′. The expression of the target gene was calculated using the 2^−ΔΔCt^ method and expressed as a fold change over that of the control.

### 2.14 Western blotting analysis

After the samples were washed in ice-cold PBS, proteins were extracted using ice-cold RIPA lysis buffer. The protein content in the supernatant was determined by conducting a BCA assay. The samples were subjected to denaturing 10% SDS-PAGE and then transferred to polyvinylidene difluoride membranes. The membrane was sealed at room temperature for 1 h with a sealing solution (5% skim milk in TBST), followed by incubation with primary antibodies, including anti-human p16 (#10883-1-AP, Proteintech, China, 1:2,000), anti-human p21 (#28248-1-AP, Proteintech, China, 1:1,000), anti-human p53 (#10442-1-AP, Proteintech, China, 1:2,000), anti-human ACSL4 (#22401-1-AP, Proteintech, China, 1:2,000), anti-human FTH1 (#ET1610-78, HUABIO, China, 1:2,000), and anti-human GPX4 (#ab231174, Abcam, United States, 1:2,000) overnight at 4°C. The blots were washed and incubated with horseradish peroxidase (HRP)-conjugated goat anti-rabbit secondary IgG antibodies (Abcam, 1:20,000) for 1 h at 37°C. Then, they were developed with an ECL detection system (Santa Cruz Biotechnology, CA, United States). GAPDH was used as a loading control. Densitometry was performed using the ImageJ software.

### 2.15 Statistical analysis

All statistical analyses were performed using GraphPad Prism 8.0 (GraphPad Software, La Jolla, CA, United States) and ImageJ. The values are expressed as the mean ± standard deviation. The normality and homogeneity of variance of the data were evaluated by the Shapiro–Wilk test. The differences in parameters between groups were determined by independent samples *t*-test. The differences in parameters among multiple groups were determined by one-way ANOVA or chi-square test. The least significant difference method was used for post-hoc multiple comparisons. All differences among and between groups were considered to be statistically significant at *p* < 0.05.

## 3 Results

### 3.1 Hyperosmotic stress induced cellular senescence in corneal epithelial cells and delayed wound healing

**Figure 1:**
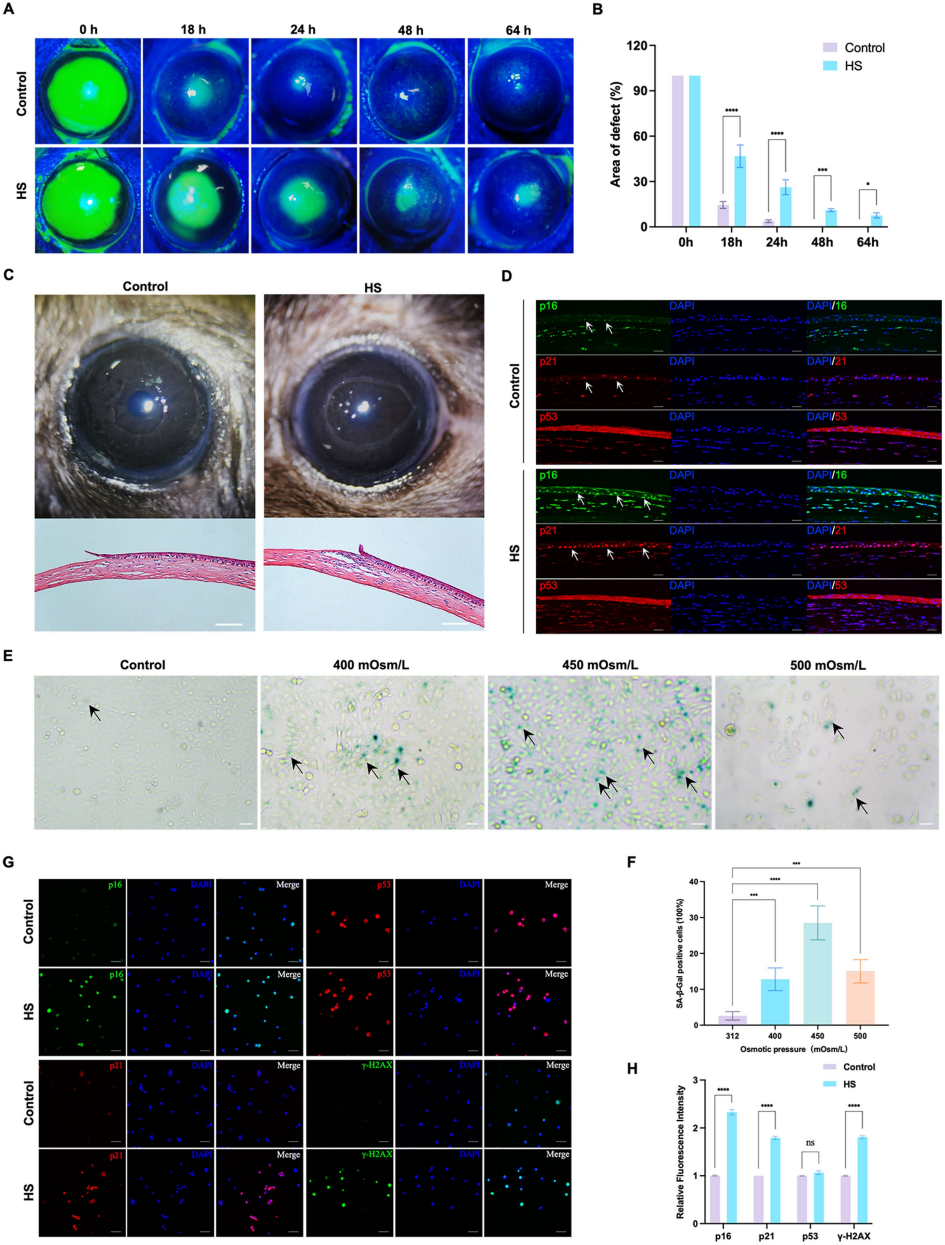
HS induced cellular senescence in cornea epithelial cells and delayed wound healing. **(A)** Representative images of corneal fluorescein staining. **(B)** Quantitative corneal epithelial defect area analysis at different times; *n* = 5. **(C)** Photos of corneal wound healing and HE staining results from 18 h after epithelial removal; scale = 100 μm. **(D)** Immunofluorescence staining of corneal epithelial senescence markers p16, p21, and p53; *n* = 5, scale = 50 μm. **(E)** SA-β-gal staining of HCE-T cells under different osmotic pressure conditions. Black arrows indicated staining positive cells; scale = 50 μm. **(F)** Quantitative analysis of SA-β-gal staining cells; *n* = 3. **(G)** Immunofluorescence staining of HCE-T cells for p16, p21, p53, and γ-H2AX; scale = 50 μm. **(H)** Quantitative analysis of immunofluorescence staining; *n* = 3. ^*^p < 0.05, ^***^p < 0.001, and ^****^p < 0.0001.

To determine the effects of HS on corneal epithelial cells, the cornea and corneal epithelial cells were treated with a hyperosmolar solution *in vivo* and *in vitro*, respectively. The healing of the corneal epithelium after injury is illustrated in Figures 1A,B. The corneal epithelium of the control group healed completely within 48 h, while defects in the epithelium were detected even 64 h after the injury was induced in the HS group. A photograph of the anterior segment showed that the corneal epithelium was defective in both groups, and the difference in corneal transparency between the groups was not significant 18 h after corneal injury. The H&E-stained sections showed that the inflammatory response was more severe in the HS group, accompanied by the infiltration of a large number of inflammatory cells (Figure 1C). The immunofluorescence results of senescence markers (p21, p16, and p53) showed that the number of p16/p21-positive corneal epithelial cells in the HS group was more than that in the control group. However, the difference in the number of p53-positive cells between the groups was not significant (Figure 1D).

*In vitro*, the HCE-T cells were exposed to isotonic (312 mOsm/L) or hypertonic (400 mOsm/L, 450 mOsm/L, and 500 mOsm/L) conditions for 120 h. The percentage of SA-β-gal-stained senescent cells was significantly higher in the 450 mOsm/L group compared to that in the control group (Figures 1E,F, *p* < 0.0001). Many cells died in the 500 mOsm/L group. Therefore, 450 mOsm/L was selected as the condition for inducing hyperosmolarity in subsequent studies. We also detected the expression of p16, p21, and p53 and the DNA damage marker γ-H2AX in HCE-T cells. Similar to the results of *in vivo* experiments, the expression of p16 and p21 in the HS group was significantly higher than that in the control group, but the difference in the expression of p53 was not significant. Moreover, the expression of γ-H2AX was also significantly higher than that in the control group (Figures 1G,H, *p* < 0.0001). These results suggested that HS can induce senescence in corneal epithelial cells.

### 3.2 UA treatment alleviated HS-induced cellular senescence *in vitro*

**Figure 2:**
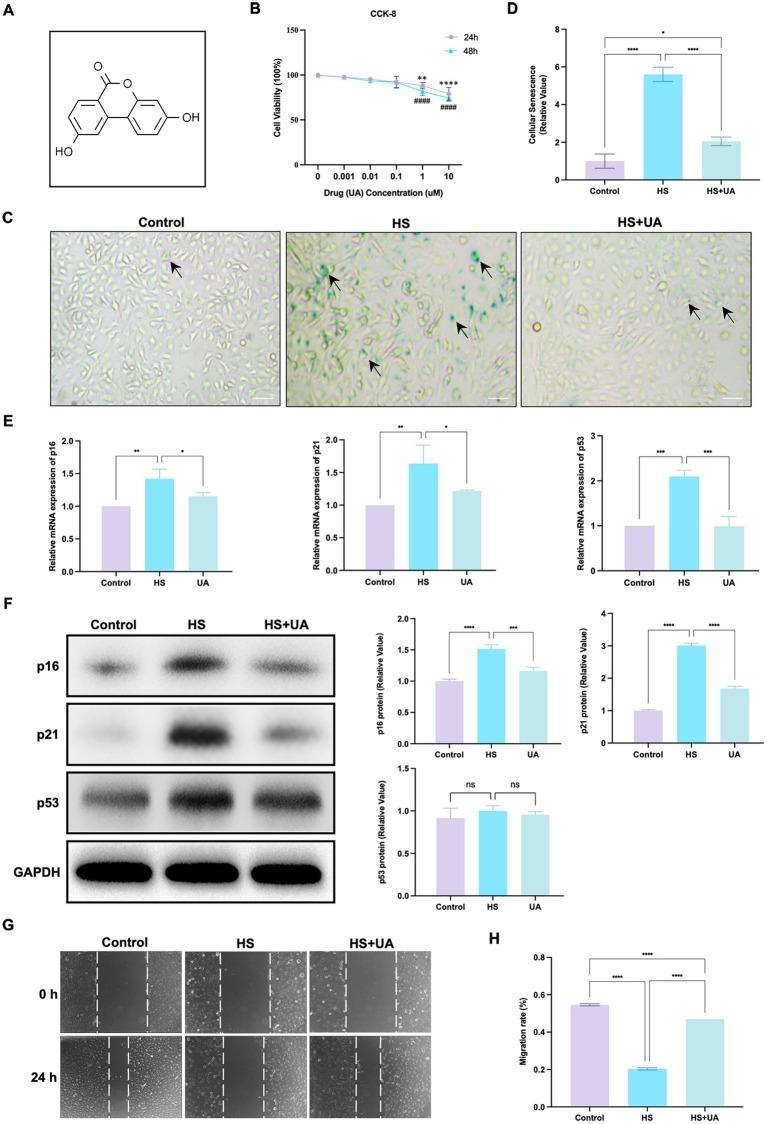
UA treatment alleviated HS-induced cellular senescence. **(A)** Molecular formula of UA. **(B)** Cell viability was tested by CCK-8 assay. **(C)** SA-β-gal staining of HCE-T cells. Black arrows indicated staining positive cells; scale = 50 μm. **(D)** Quantitative analysis of SA-β-gal staining results. **(E)** Relative mRNA expression of p16, p21, and p53. **(F)** Representative western blot images and quantitative analysis of brand intensity. **(G)** The scratch test and **(H)** quantitative analysis. *n* = 3, ^*^p < 0.05, ^**^p < 0.01, ^***^p < 0.001, and ^****^p < 0.0001.

We conducted relevant experiments to determine whether UA can rescue HCE-T cells under hyperosmotic conditions (Figure 2A). First, we treated HCE-T cells with 0.001, 0.01, 0.1, 1, and 10 μM UA for 24 h and 48 h, respectively. When the concentration was higher than 0.1 μM, the cell viability decreased significantly (Figure 2B, *p* < 0.01). Therefore, 0.1 μM UA was used in subsequent studies. There was no significant difference in SA-β-gal staining results between the cells treated with UA alone and the control group (Supplementary Figure S1). HCE-T cells were treated with UA for 24 h and then exposed to high-osmolarity conditions for 120 h. The percentage of SA-β-gal-stained cells increased significantly after hypertonic stimulation compared to that in the control group. Moreover, UA treatment decreased the percentage of SA-β-gal-stained cells (Figures 2C,D, *p* < 0.0001).

We also assessed mRNA and protein levels of cell senescence-relevant markers. The results showed that the level of expression of the p16, p21, and p53 mRNAs in HCE-T cells was significantly upregulated under hypertonic conditions compared to that in the control group, and this upregulation was reversed after UA treatment (Figure 2E). The results of western blotting assays showed that the expression of p16 and p21 was consistent with RT-qPCR, but no significant difference was found in p53 protein levels (Figure 2F). These findings indicated that UA can alleviate the senescence of HCE-T cells caused by HS.

We further assessed cell migration capacity. The result showed that the migration ability of HCE-T cells decreased significantly under HS conditions (*p* < 0.0001). After UA treatment, cell migration ability improved considerably (Figures 2G,H), which suggested that the accumulation of senescent cells caused by HS can inhibit the migration of cells, and UA treatment can reverse the delay in cell migration.

### 3.3 UA treatment can alleviate delayed corneal epithelial wound healing under hyperosmotic conditions

**Figure 3:**
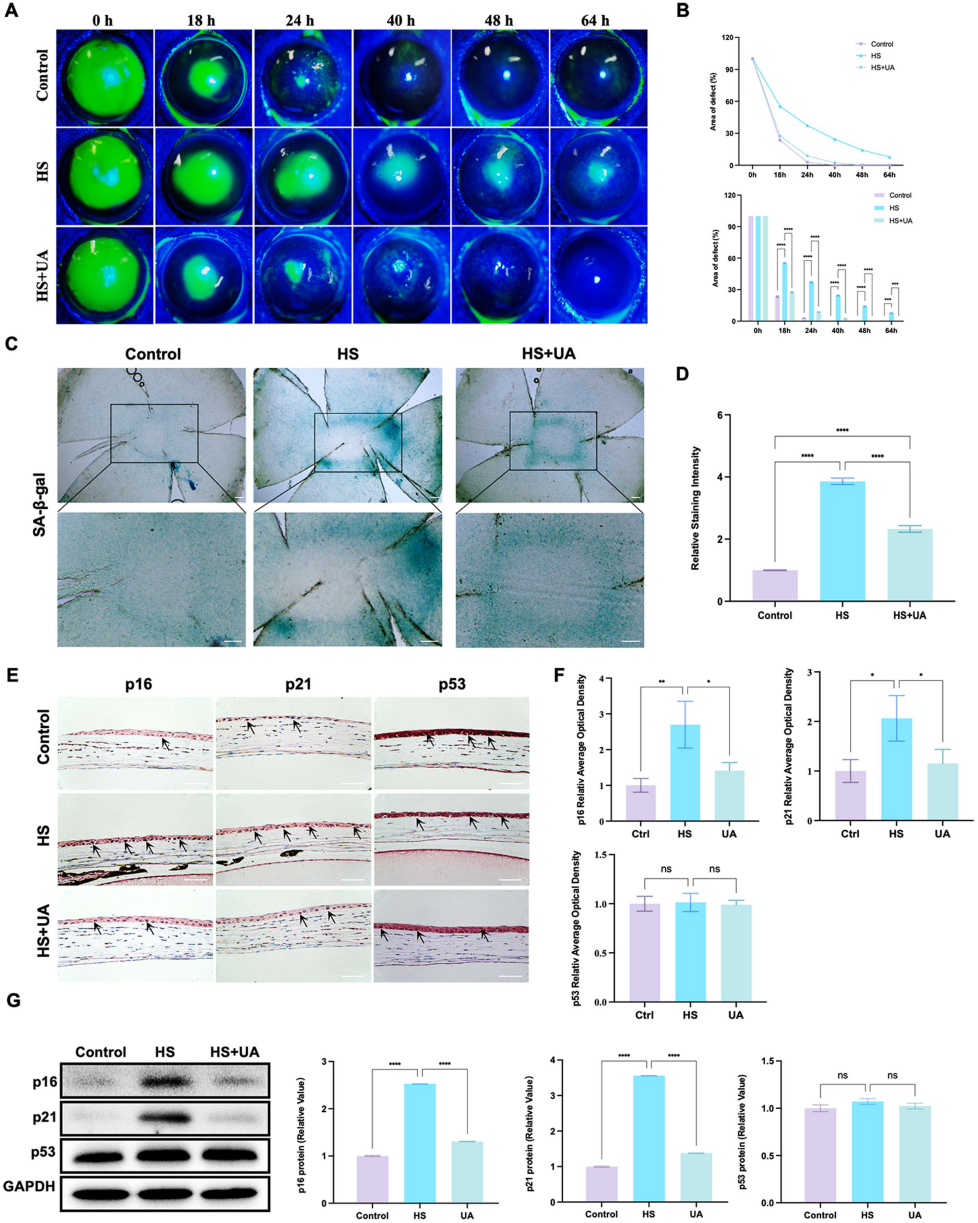
UA improved the symptoms of delayed healing of corneal epithelial wounds induced by HS in mice. **(A)** Representative images of corneal fluorescein sodium staining. **(B)** The trend of corneal epithelial defect areas at different follow-up times. **(C)** SA-β-gal staining of flat-mounted corneas; scale = 200 μm. **(D)** Quantitative analysis of SA-β-gal staining results. **(E)** Immunohistochemical staining results of p16, p21, and p53 in mouse corneal epithelium; scale = 100 μm. **(F)** Quantitative analysis of immunohistochemical results. **(G)** Representative western blot images and quantitative analysis of brand intensity. *n* = 3, ^*^p < 0.05, ^**^p < 0.01, ^***^p < 0.001, and ^****^p < 0.0001.

As the application of cell lines in simulating the physiological characteristics of corneal epithelial cells has some limitations, we established an animal model of corneal epithelial cell senescence by continuous eye intervention with hypertonic saline and further investigated the anti-senescence effects of UA *in vivo*. The corneal epithelium was carefully removed after intervention with the hypertonic solution, and the healing of corneal epithelium was observed at 0, 18, 24, 40, 48, and 64 h, respectively (Figure 3A). In the control and UA groups, the corneal epithelium healed completely within 48 h. However, the corneal epithelium of the HS group neither healed completely at 48 h nor at 64 h.

We also quantified the area of corneal epithelial defect in the three groups at different times. The results showed that the average defect area of the HS group was 55.37% 18 h after surgery and that of the UA intervention group was 27.55%, which was significantly smaller than that of the HS group (*p* < 0.0001). At 48 h after surgery, the defect area of the HS group was 14.18%. In contrast, the epithelium was fully healed in the UA group, similar to that in the control group (Figure 3B). Therefore, HS can delay the healing of corneal epithelium in mice, and the wound healing time is significantly shortened after UA treatment. These findings suggested that UA can relieve the symptoms of poor corneal epithelial wound healing caused by HS.

We also found that continuous hypertonic stimulation resulted in the accumulation of many SA-β-gal-stained positive cells around the central corneal epithelial wound, whereas, the number of senescent cells significantly decreased in the UA group (Figures 3C,D, *p* < 0.0001). We also examined the expression and distribution of senescence markers in the cornea. The results of immunohistochemical assays showed that the level of expression of p16 and p21 in the epithelium increased considerably in the HS group, and their levels recovered significantly after UA treatment (Figures 3E,F). Similarly, the level of expression of the p16 and p21 proteins in the HS group was significantly upregulated, and UA treatment decreased their expression (Figure 3G). Thus, a decrease in the expression of p16 and p21 and a decrease in the positive rate of SA-β-gal staining indicated that UA can inhibit HS-induced senescence of corneal epithelial cells. Thus, the effect of UA on the poor healing of corneal epithelial wounds might be related to its anti-senescence effect.

### 3.4 Ferroptosis was involved in cell senescence caused by hyperosmolarity

**Figure 4:**
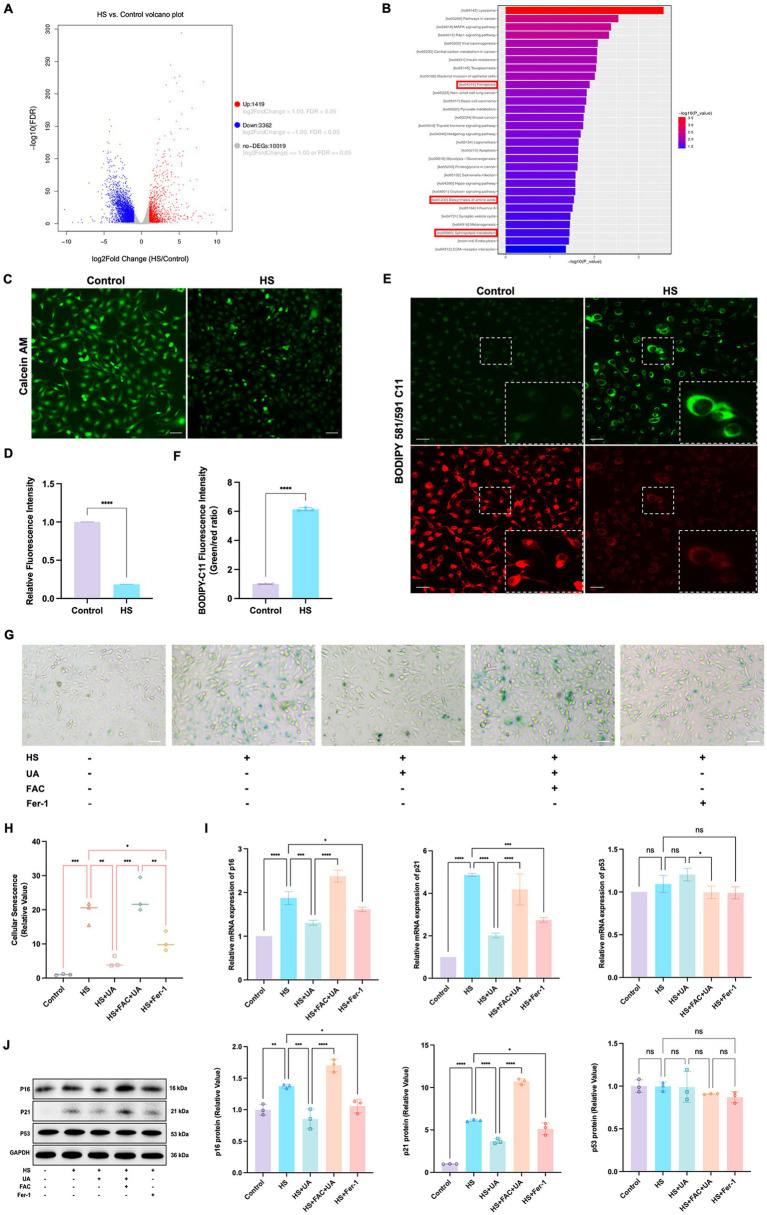
Ferroptosis was involved in cell senescence caused by hyperosmolarity. **(A)** Volcano plot shows significant genes that altered in the HS group and control group. **(B)** KEGG pathway enrichment bar chart of significantly different genes in the HS group and control group. **(C)** Representative images of intracellular labile iron indicated by calcein-AM assay; scale = 50 μm. **(D)** Quantitative analysis of calcein fluorescence density. **(E)** Representative images of LPO indicated by BODIPY-C11 probe; scale = 50 μm. **(F)** Quantitative analysis of the fluorescence intensity of oxidized and non-oxidized BODIPY-C11. **(G)** SA-β-gal staining of HCE-T cells; scale = 50 μm. **(H)** Quantitative analysis of SA-β-gal staining cells. **(I)** Relative mRNA expression of p16, p21, and p53. **(J)** Representative western blot images and quantitative analysis of brand intensity. *n* = 3, ^*^p < 0.05, ^**^p < 0.01, ^***^p < 0.001, and ^****^p < 0.0001.

To further investigate the mechanisms underlying HS-induced cell senescence, we conducted transcriptome sequencing analysis on cells from the HS and control groups. The results revealed that the genes differentially expressed between the groups were primarily enriched in signaling pathways related to ferroptosis, amino acid metabolism, and phospholipid metabolism (Figures 4A,B). Ferroptosis is closely related to cell metabolism and cell fate, and we further examined the involvement of ferroptosis in cell senescence caused by HS. The results of calcein AM staining showed that the intracellular labile iron content in the HS group was significantly higher than that in the control group (Figures 4C,D). Ferroptosis is closely related to an increase in oxidative stress, especially lipid peroxidation. Our results indicated that the level of LPO in HCE-T cells was significantly higher in the HS group than in the control group (Figures 4E,F), manifesting as a significant increase in the fluorescence intensity of oxidized BODIPY-C11 and a relative decrease in the fluorescence intensity of non-oxidized BODIPY-C11. These results indicated that HS treatment can induce ferroptosis in HCE-T cells.

To further elucidate the role of ferroptosis in the senescence of HCE-T cells, we conducted an intervention experiment with the ferroptosis inducer ferric ammonium citrate (FAC) and the ferroptosis inhibitor Ferrostatin-1 (Fer-1). We designated the condition of treating HCE-T cells with 500 μmol/L FAC for 48 h as a positive control for inducing ferroptosis (Supplementary Figures S2A,B). The highest number of SA-β-gal positive cells was recorded in the FAC + UA group, while compared to the HS group, the number of SA-β-gal positive cells in the UA and Fer-1 groups decreased significantly (Figures 4G,H). Additionally, the level of expression of the mRNAs and proteins of p16 and p21 in the UA and Fer-1 groups were lower than their respective levels in the HS group (Figures 4I,J). This implied that FAC-induced ferroptosis can reverse the anti-senescence effects of UA, and conversely, inhibiting ferroptosis can alleviate the senescence of HCE-T cells. Our results also showed that there were no significant differences in p53 levels among all groups, similar to our previous findings, indicating that HS-induced senescence and ferroptosis may be independent of p53.

### 3.5 UA intervention inhibited ferroptosis induced by hyperosmolarity *in vitro*

**Figure 5:**
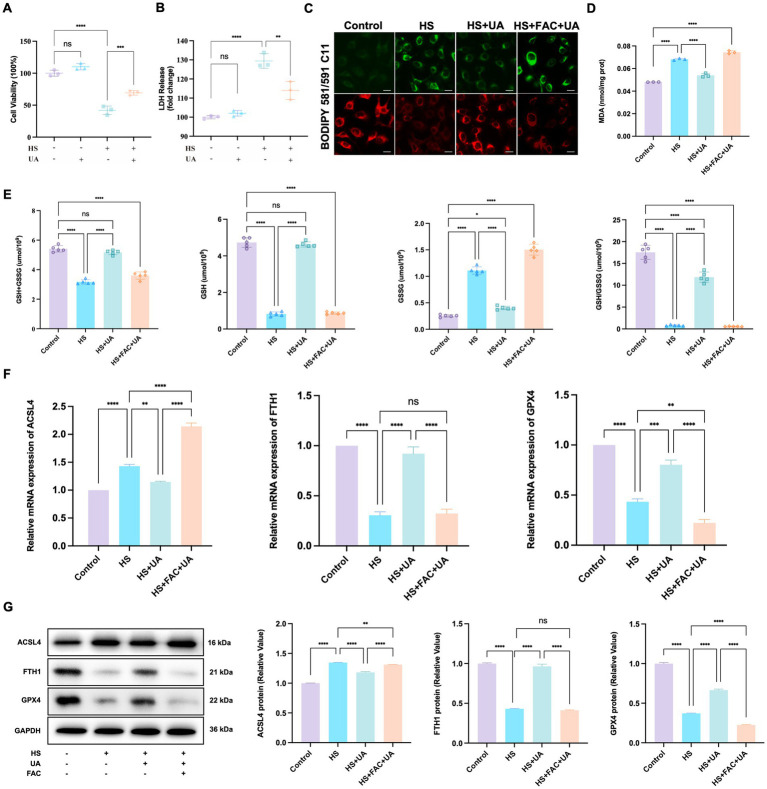
UA intervention inhibited the ferroptosis process induced by hyperosmolarity. **(A)** Cell viability was tested by CCK-8 assay. **(B)** Cytotoxicity was tested by LDH assay. **(C)** Representative images of LPO indicated by BODIPY-C11 probe; scale = 20 μm. **(D)** MDA levels in HCE-T cells. **(E)** The levels of GSH + GSSG, GSH, GSSG, and GSH/GSSG in HCE-T cells. **(F)** Relative mRNA expression of ACSL4, FTH1, and GPX4. **(G)** Representative western blot images and quantitative analysis of brand intensity. *n* = 3, ^*^p < 0.05, ^**^p < 0.01, ^***^p < 0.001, and ^****^p < 0.0001.

As ferroptosis was involved in cell senescence caused by hyperosmolarity, we investigated whether UA alleviated cell senescence through ferroptosis and determined its exact regulatory mechanisms. First, the results of the CCK-8 assay showed that UA treatment strongly inhibited the HS-induced decrease in the viability of HCE-T cells (Figure 5A). These results were similar to those of the LDH release assay, which showed that HS exposure significantly increased the cell toxicity of HCE-T compared to the control group, while UA treatment effectively rescued HCE-T (Figure 5B). The fluorescence intensity of oxidized BODIPY-C11 significantly increased in the HS group compared to the fluorescence intensity recorded in the control group (Figure 5C). After UA treatment, the fluorescence intensity of non-oxidized BODIPY-C11 increased, which indicated that UA can significantly decrease the level of expression of LPO. However, when co-treated with FAC, the level of LPO increased significantly and reversed the effects of UA.

We also measured the levels of MDA and GSH related to oxidative stress. We found that HS stimulated the production of MDA in HCE-T cells relative to MDA production in the control group. In contrast, UA decreased intracellular MDA levels (*p* < 0.0001, Figure 5D). GSH metabolism strongly influences redox metabolism and ferroptosis. It serves as an electron donor of GPX4, and a decrease in GSH levels can influence the antioxidant capacity and lipid metabolism of cells. Our results showed that the total GSH level (GSH + GSSG) of HCE-T cells exposed to HS decreased. Among them, the GSSG level increased significantly, while the GSH/GSSG and GSH levels were considerably lower than those in the control group (*p* < 0.0001). After UA treatment, the GSSG level in HCE-T cells decreased, and the GSH/GSSG and GSH levels were significantly restored (*p* < 0.0001, Figure 5E). Moreover, additional treatment of FAC caused the levels of GSH/GSSG and GSH to decrease again, eliminating the inhibitory effect of UA on ferroptosis. Therefore, the inhibition of HS-induced ferroptosis in HCE-T cells by UA is associated with a decrease in oxidative stress levels.

Ferroptosis involves multiple molecules, among which FTH1 and GPX4 are negatively correlated with the occurrence of ferroptosis, while ACSL4 is positively correlated with ferroptosis. To determine the molecular mechanism of action of these molecules, we conducted RT-qPCR and western blotting assays on the aforementioned key molecules. The results of RT-qPCR assays showed that HS exposure upregulated the expression of ACSL4 in HCE-T cells but downregulated the expression of FTH1 and GPX4. In the UA group, the results were the opposite. The level of expression of ACSL4 was downregulated, while the expression of FTH1 and GPX4 mRNAs was significantly upregulated (Figure 5F). The results of western blotting assays matched those of the RT-qPCR assays (Figure 5G). These results indicated that UA inhibits HS-induced ferroptosis in cells by upregulating GPX4 and FTH1 and suppressing ACSL4.

## 4 Discussion

**Figure 6:**
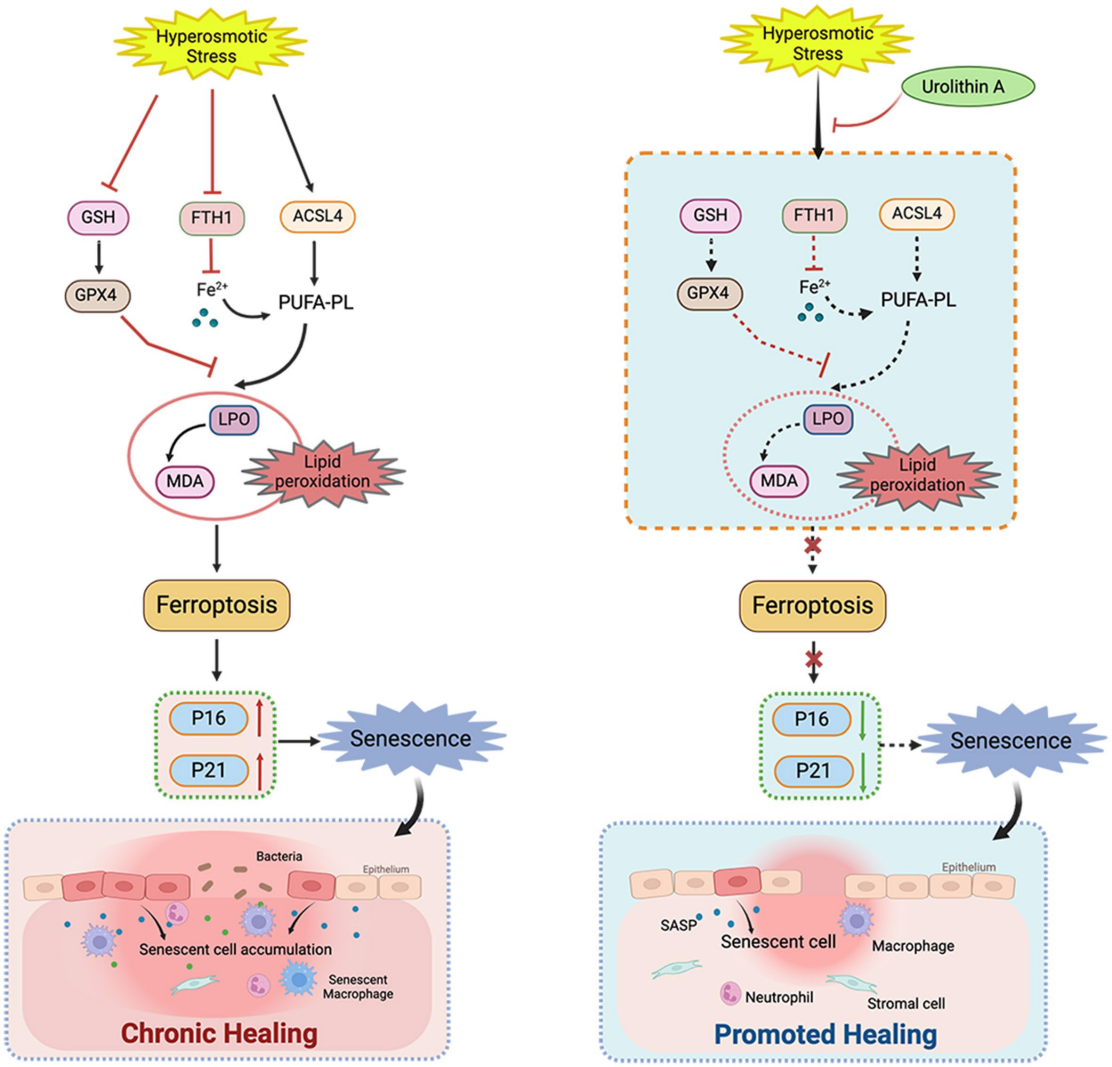
Schematic diagram of the mechanism of UA on corneal epithelial cells. UA alleviated HS-induced corneal epithelial cell senescence by inhibiting ferroptosis and thus promoted corneal epithelial healing.

In this study, we analyzed the role of cell senescence in damage caused by HS to the corneal epithelium and determined the role of UA in HS-related cell senescence. We found that continuous hypertonic stimulation can cause the senescence of corneal epithelial cells and significantly delay wound healing. UA treatment can decrease the proportion of SA-β-gal staining positive cells, downregulate the expression of p16 and p21, and promote cell migration. We also found that the senescence of HCE-T cells is regulated by ferroptosis, and inhibiting ferroptosis can alleviate the senescence of HCE-T cells (Figure 6).

It is worth noting that the expression of p21 and p16 in corneal epithelial cells after HS treatment was significantly up-regulated, while p53 had no significant effect. When cells are stimulated by external stress, DNA-damage response leads to the activation of p53/p21 and p16 pathways, blocking the cell cycle and entering the cell senescence process. p21 is a downstream gene of p53 and its expression is regulated by p53. However, p21 has been found to induce premature senescence in p53-deficient H1299 cells (24). In senescent embryonic cells, p21 is regulated by TGF-β/SMAD and PI3K/ FOXO signaling pathways, but not by p53 (25). Englund et al. (26) found that p21 alone was sufficient to drive the cellular senescence process, leading to aging and functional decline of skeletal muscle cells. In addition, although the silencing of p53 gene can reduce the expression of p21, the expression of p21 can be significantly upregulated in cells that simultaneously knocked down the histone methyltransferase SETD8, indicating that the change of p21 is completely independent of p53 (27). Therefore, we speculate that HS may promote corneal epithelial cell senescence in a non-p53-dependent pathway.

Cellular senescence strongly influences wound healing (28). Senescent lung fibroblasts induce cell cycle arrest in the G2/M phase in alveolar epithelial cells, leading to abnormal wound repair and re-epithelialization (29). Samdavid Thanapaul et al. (11) found that in an irradiation-induced skin aging model, skin fibroblast aging delayed wound healing. Chia et al. (30) found that the level of expression of p21 increased significantly during wound healing in the elderly, and abnormal aging indicators explained the abnormal skin repair ability. Additionally, removing senescent cells can alleviate poor wound healing and delay age-related diseases. By inhibiting high glucose-induced cellular senescence, collagen deposition and the expression of α-SMA can be accelerated to promote the healing of diabetic wounds (12). Saul et al. (31) found that the intermittent administration of the senescent cell-eliminating drug dasatinib combined with quercetin decreased the senescence markers in the fracture callus of adult mice, significantly reduced the fracture healing time, and promoted tissue repair. We found that continuously exposing the corneal epithelium of mice to hypertonic conditions resulted in a significant delay in wound healing, and many senescent cells were observed at the edge of the corneal epithelial wound. After UA treatment, the level of expression of p16 and p21 in HCE-T cells and mouse corneal epithelial cells were significantly downregulated, and the proportion of SA-β-gal-stained cells decreased, which confirmed that corneal epithelial healing and damage repair could be promoted by alleviating cell senescence.

The RNA-seq results also indicated that ferroptosis was significantly dysregulated. Ferroptosis is immunogenic and causes cell damage along with a cascade of amplified inflammatory reactions, aggravating the damage in surrounding tissues (32). Some studies have shown that ferroptosis and cellular senescence are correlated (33–36). Several studies have reported abnormalities in iron metabolism in cells in many organisms (e.g., fruit flies, rodents, humans, etc.), especially in the brain, where excess iron can lead to neurodegenerative diseases, such as Alzheimer’s and Parkinson’s disease (37–39). Sun et al. (40) found that erastin, a ferroptosis inducer, increased the percentage of SA-β-gal positive cells by depleting GSH, which indicated that GSH depletion can induce ferroptosis in retinal pigment epithelial cells while also leading to cell growth arrest and premature cell senescence. In this study, HCE-T treated with FAC showed the highest number of SA-β-gal positive cells. However, compared to the HS group, the number of SA-β-gal positive cells in the UA and Fer-1 groups decreased significantly. Moreover, the expression of p16 and p21 in the UA and Fer-1 groups was lower than that in the HS group, which implied that FAC-induced ferroptosis reversed the anti-senescence effects of UA, whereas, inhibiting ferroptosis alleviated the senescence of HCE-T cells.

Ferroptosis is closely related to an increase in oxidation levels, especially lipid peroxidation levels (41). Lou et al. (42) showed that UA can reduce the lung dry weight-to-wet weight ratio *in vivo*, decrease the production of ROS and MDA and the depletion of GSH, and thus, effectively alleviate LPS-induced ferroptosis and acute lung injury in mice. By estimating the levels of LPO, MDA, and GSH related to oxidative stress, we found that UA significantly reduced the susceptibility of HCE-T cells to the induction of HS and the levels of LPO, MDA, and GSSG. It also significantly recovered GSH, which showed a cytoprotective effect. These findings were also similar to those reported by Xu et al. (43), who showed that 1,25(OH)_2_D_3_ can downregulate senescence-related genes, MDA, and 4-hydroxynonenal and upregulate GSH by inhibiting D-gal-induced ferroptosis, thus delaying osteoblast senescence and preventing osteoporosis. Therefore, UA treatment reduced the level of oxidative stress in HCE-T cells and inhibited ferroptosis. It also reversed the HS-induced upregulation of ACSL4 and downregulation of FTH1 and GPX4. This finding further supported the speculation that UA can delay HCE-T cell senescence by inhibiting ferroptosis.

However, this study had many limitations. First, the studies on the mechanism of action were mainly *in vitro*, and whether ferroptosis inhibition can inhibit cell senescence *in vivo* needs to be confirmed. Second, studies on macroscopic ferroptosis were preliminary. Thus, the role of specific molecules in this process needs to be investigated through gene editing. Finally, several causative agents account for cell senescence, and whether the conclusion of this study is consistent with the cell senescence caused by hypoxia, high sugar levels, and other stimuli needs to be confirmed by constructing larger and more robust models.

## 5 Conclusion

To summarize, our study suggested that cell senescence plays a crucial role in the healing of the corneal epithelium. As UA can alleviate the senescence of HCE-T cells by inhibiting ferroptosis, it can be used to develop effective strategies for treating poor corneal wound healing.

## Data Availability

The data presented in the study are deposited in the NCBI repository at https://www.ncbi.nlm.nih.gov/bioproject/1154417, accession number PRJNA1154417. Further inquiries can be directed to the corresponding author.
