# Supplementary figures and images for: Urolithin A alleviates cell senescence by inhibiting ferroptosis and enhances corneal epithelial wound healing
Source: Front Med (Lausanne). 2024 Sep 16;11:1441196. doi: 10.3389/fmed.2024.1441196 (PMC11439666; doi:10.3389/fmed.2024.1441196)

## B vs. A volcano plot

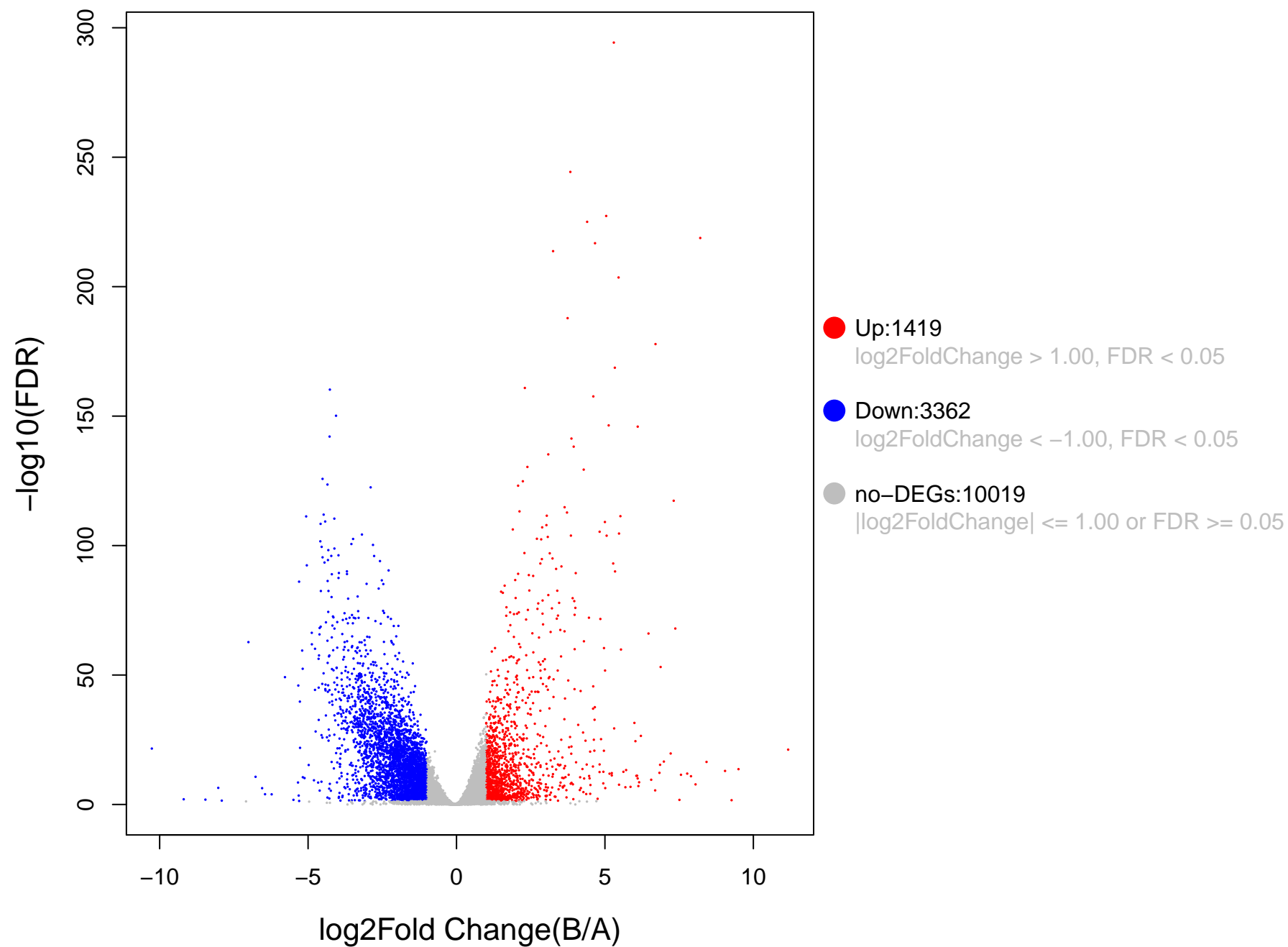

Supplement: Supplementary file 1 [file Data_Sheet_1.zip › RNA-seq data/02.expldiff_visualisation/Group.HS_vs_CTRL.VolcanoPlot.pdf]

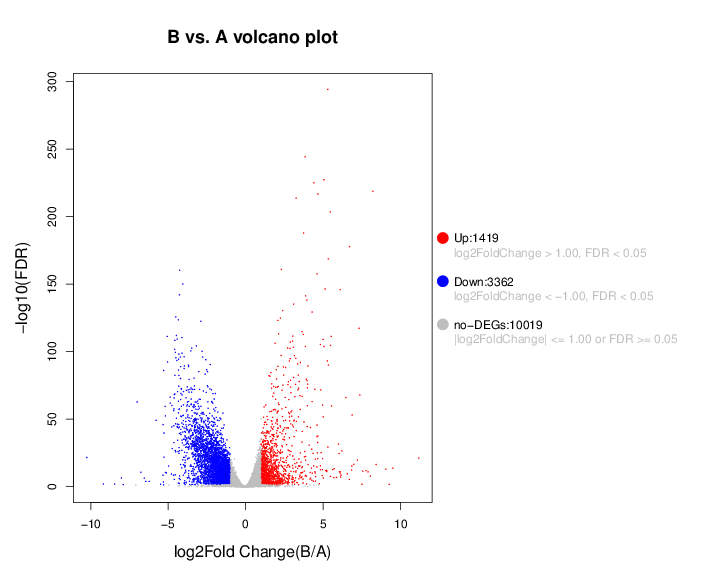

Supplement: Supplementary file 1 [file Data_Sheet_1.zip › RNA-seq data/02.expldiff_visualisation/Group.HS_vs_CTRL.VolcanoPlot.png]

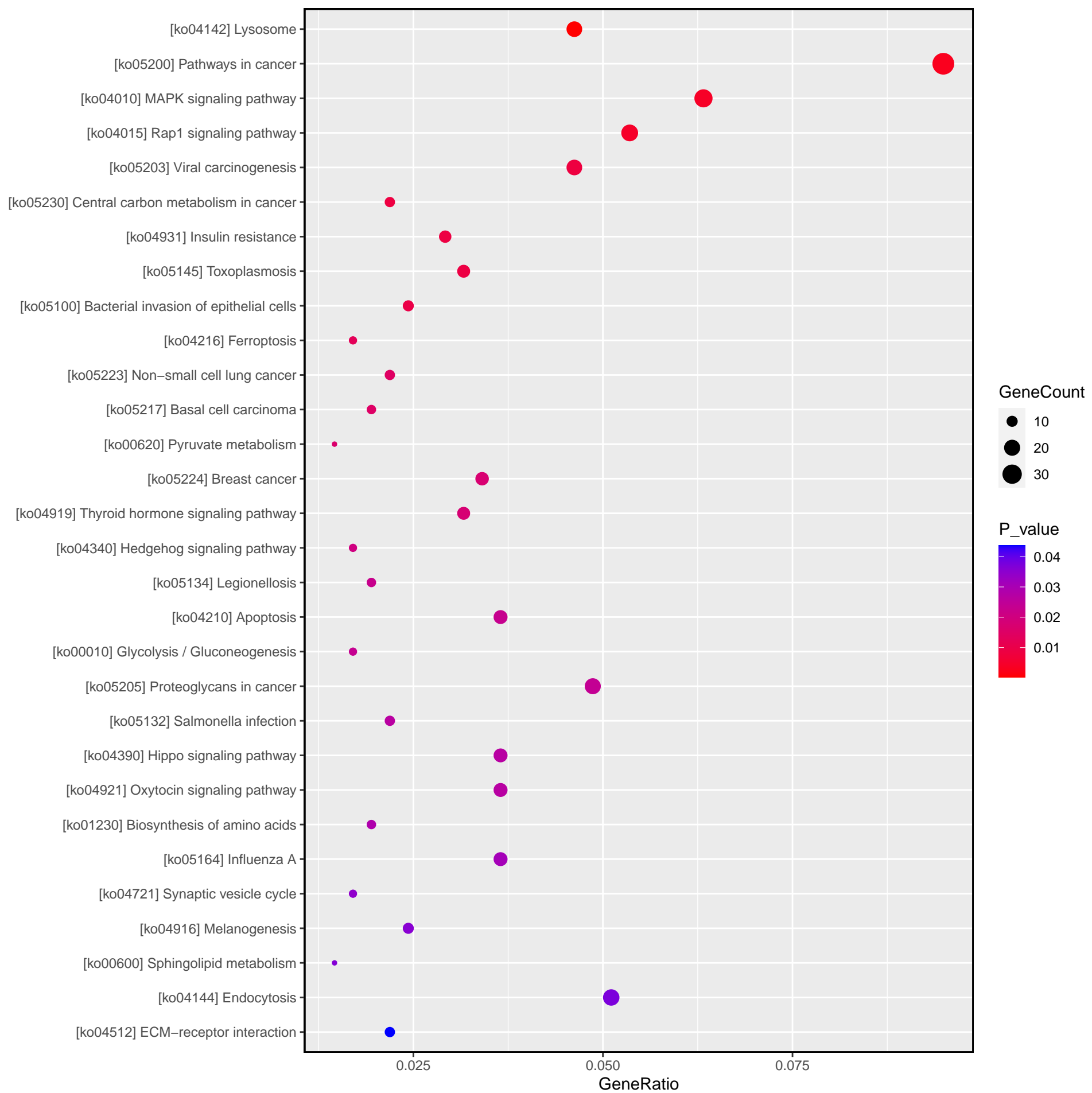

Supplement: Supplementary file 1 [file Data_Sheet_1.zip › RNA-seq data/03.expldiff_enrichment_KEGG/dotplot/Group.HS_vs_CTRL.de_all.KEGG_ALL.dotplot.pdf]

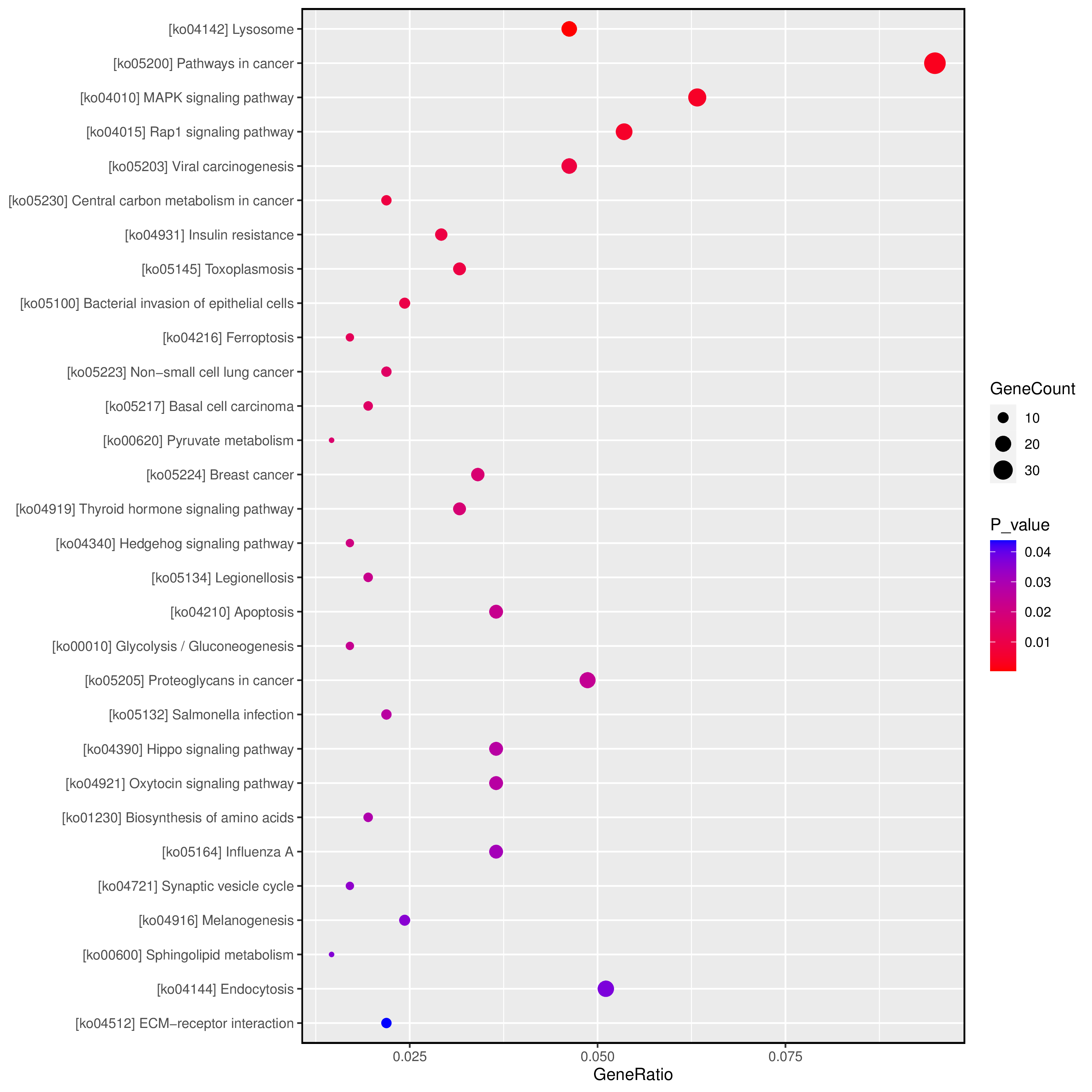

Supplement: Supplementary file 1 [file Data_Sheet_1.zip › RNA-seq data/03.expldiff_enrichment_KEGG/dotplot/Group.HS_vs_CTRL.de_all.KEGG_ALL.dotplot.png]

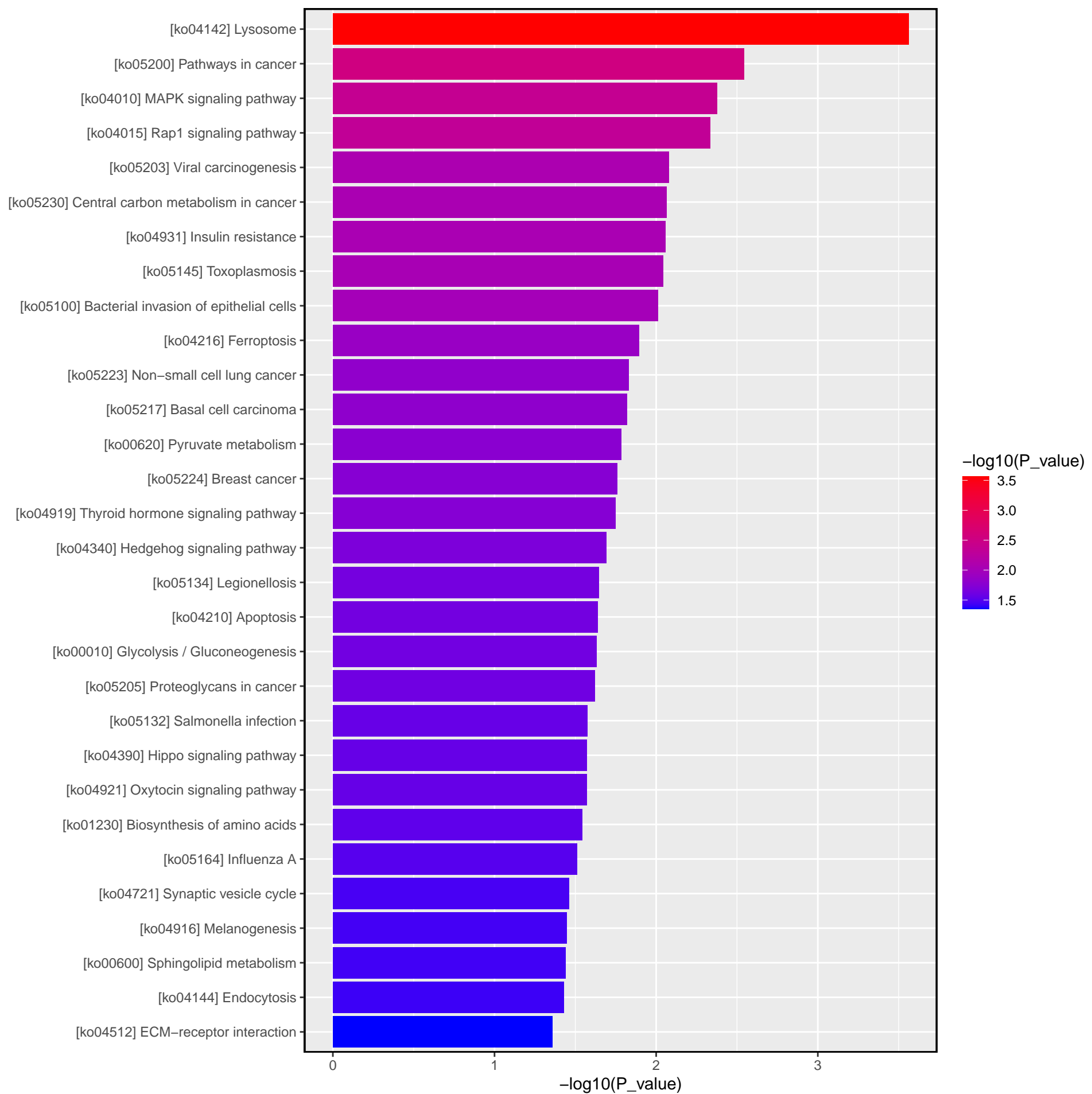

Supplement: Supplementary file 1 [file Data_Sheet_1.zip › RNA-seq data/03.expldiff_enrichment_KEGG/barplot/Group.HS_vs_CTRL.de_all.KEGG_ALL.barplot.pdf]

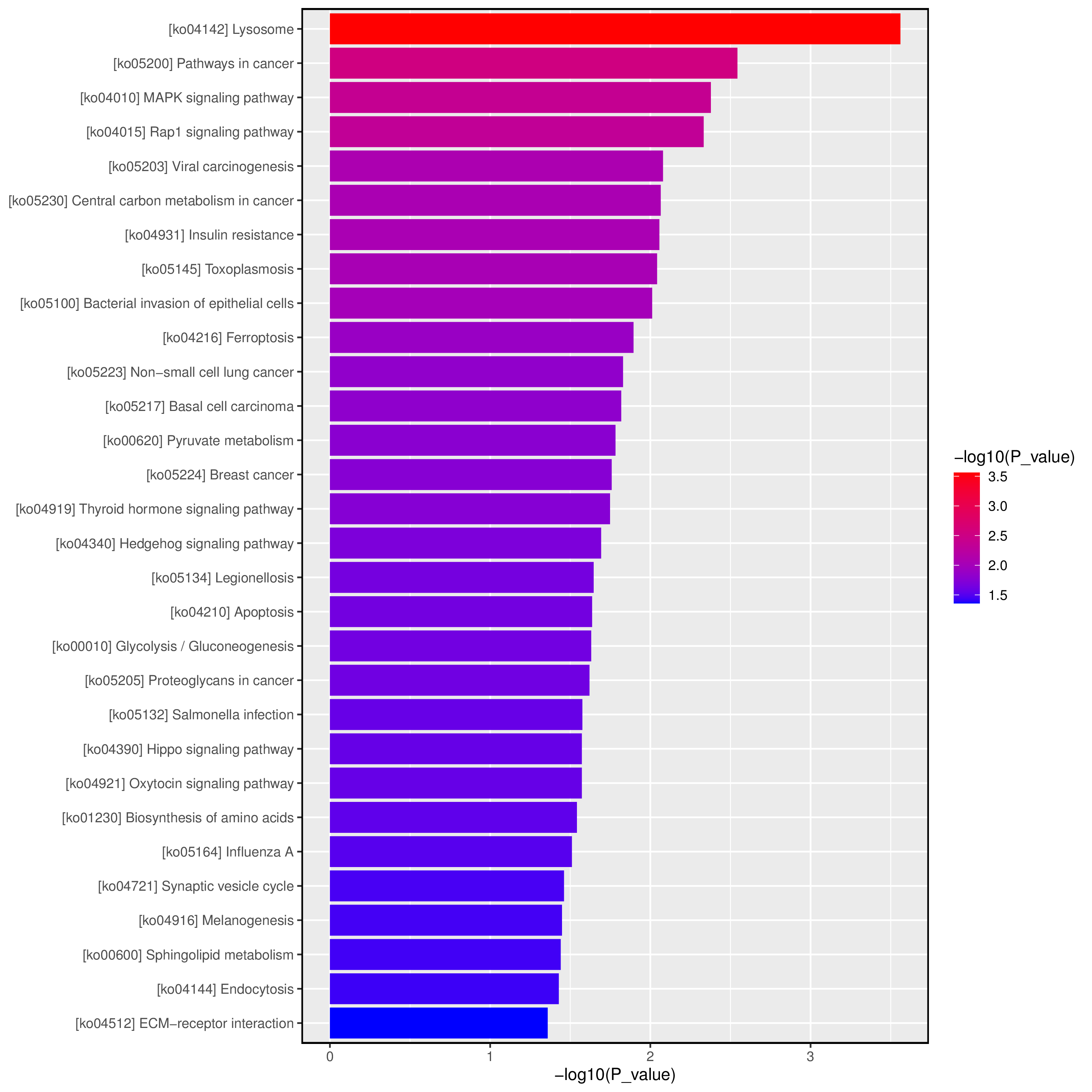

Supplement: Supplementary file 1 [file Data_Sheet_1.zip › RNA-seq data/03.expldiff_enrichment_KEGG/barplot/Group.HS_vs_CTRL.de_all.KEGG_ALL.barplot.png]
