# Supplementary material for: Urolithin A alleviates cell senescence by inhibiting ferroptosis and enhances corneal epithelial wound healing
Source: Front Med (Lausanne). 2024 Sep 16;11:1441196. doi: 10.3389/fmed.2024.1441196 (PMC11439666; doi:10.3389/fmed.2024.1441196)
Supplement: Supplementary file 2 [file Data_Sheet_2.docx]

Supplementary Material

# Supplementary Figures

## Supplementary Figures 1


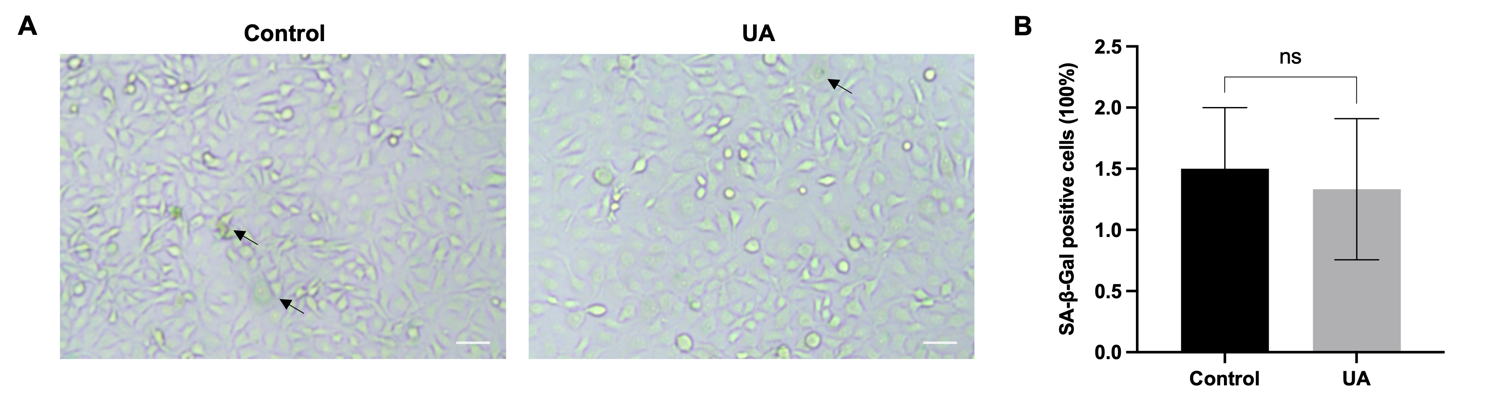


**Supplementary Figure 1.** (A) SA-β-Gal staining of HCE-T cells. Black arrows indicated staining positive cells; scale = 50 um. (B) Quantitative analysis of SA-β-Gal staining results. n = 3, ns = not significant

## Supplementary Figures 2


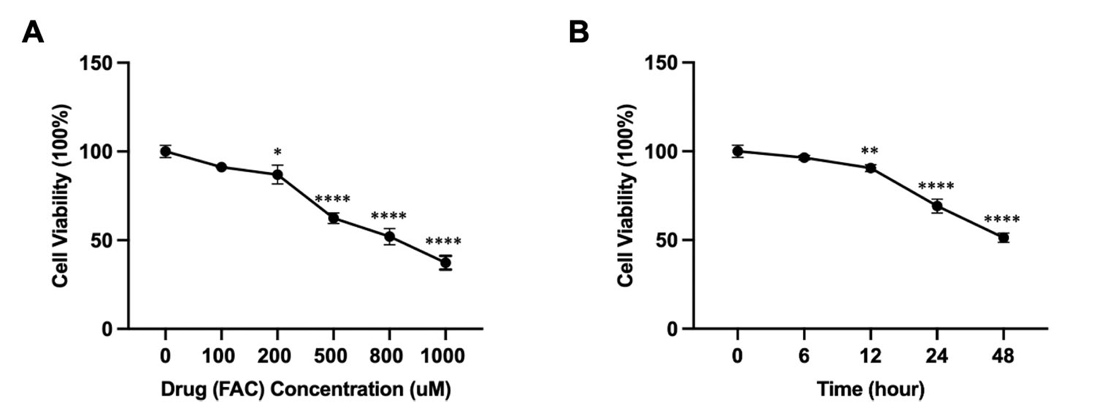


**Supplementary Figure 2.** FAC inhibited the proliferation of HCE-T cells. (A) HCE-T cells were treated with various concentrations of FAC at 37 °C for 24 h; (B) HCE-T cells were incubated with 500 μmol/L FAC for 0, 6, 12, 24, and 48 h. The effects on cell proliferation were examined by CCK-8 assay. n = 3，*p < 0.05，**p < 0.01, ****p < 0.0001.
